# The HIV Cascade of Care and Service Utilisation at Sex Work Programmes Among Female Sex Workers in South Africa

**DOI:** 10.1007/s10461-022-03616-6

**Published:** 2022-03-05

**Authors:** Maya Jaffer, Nicola Christofides, Khuthadzo Hlongwane, Kennedy Otwombe, Minja Milovanovic, Kathryn L. Hopkins, Mokgadi Matuludi, Venice Mbowane, Fareed Abdullah, Glenda Gray, Rachel Jewkes, Jenny Coetzee

**Affiliations:** 1 Perinatal HIV Research Unit, Faculty of Health Sciences, University of the Witwatersrand, Johannesburg, South Africa; 2 School of Public Health, University of the Witwatersrand, Johannesburg, South Africa; 3 African Potential Management Consultancy, Kyalami, South Africa; 4 Office of AIDS and TB Research, South African Medical Research Council, Pretoria, South Africa; 5 Office of the President, South African Medical Research Council, Cape Town, South Africa

**Keywords:** Cascade of care, Sex worker, HIV, Service offering, Program, South Africa

## Abstract

Female sex workers (FSWs) in South Africa experience a uniquely high prevalence of HIV. We describe the HIV cascade of care (CoC) in FSWs in South Africa, and explored service utilisation at sex work programmes. A cross-sectional, study enrolled FSWs across 12 sites in South Africa. Participants were recruited using chain-referral method. Inclusion criteria: ≥ 18 years, cis-gender female, sold/transacted in sex, HIV positive. 1862 HIV positive FSWs were enrolled. 92% were known positive, 87% were on antiretroviral treatment (ART). Of those on ART, 74% were virally suppressed. Younger FSWs were significantly less likely to be on ART or virally suppressed. Female sex workers using HIV services from specialised programs were 1.4 times more likely to be virally suppressed than non-program users. The pre-COVID-19 pandemic HIV CoC amongst FSWs in South Africa shows striking improvement from previous estimates, and approaches achievement of 90:90:90 goals.

**Supplementary Information:**

The online version contains supplementary material available at 10.1007/s10461-022-03616-6.

## Introduction

Female sex workers (FSWs) in South Africa are disproportionately affected by HIV infection, with a reported prevalence ranging between 40 and 80% within major cities [1–5]. In recent years, the National Department of Health (NDoH) has focused attention on sex worker vulnerability to HIV, through the implementation of the National Sex Worker HIV Plan in 2016 [6], with an overdue update to the SA National Sex Worker HIV TB STI Plan (2019–2022) [7]. The plan involved scaling-up of specialised, sex work-friendly health programmes with the aim of achieving the World Health Organization’s (WHO) 90:90:90 cascade of care (CoC) targets. This CoC requires 90% of HIV-positive sex workers to know their status, of which 90% should be initiated on antiretroviral treatment (ART); and of those, 90% should reach viral suppression [6].

Challenges towards the provision of HIV services provided amongst sex worker populations are well-documented, with testing and treatment outcomes inferior to those seen within the general population [8, 9]. The effect of stigma on sex worker health-seeking behaviour is critical, with global and local studies demonstrating a link between fear of and experienced discrimination, particularly from health care workers (HCWs), and a lower willingness to engage in health services [10–16]. Other barriers include high sex worker mobility [14, 17], non-disclosure of HIV status to non-paying partners [18], and the prevalence of food and financial insecurity in the population [9]. Sociodemographic factors are also determinants of the HIV CoC, with research in South Africa and other countries in the region showing younger FSWs are less likely to to be aware of an HIV-positive status, to be taking ART, or to be virally suppressed [19, 20]. Cross-border immigrant sex workers in South Africa have also been found to have lower contact with health facilities [21].

There is strong evidence of improved care for sex workers through specialised sex work programmes as opposed to facilities geared towards the general population. Peer-implemented outreach is associated with improved uptake of HIV services [20, 22], and this evidence informed the South African National Sex Worker HIV Plan to emphasise a peer-led approach [6]. Qualitiative research supports a preference for sex worker-friendly clinics in which interacting with sensitised staff is a motivator to attend services [16, 17, 23, 24]. Access may also be facilitated by after-hours and mobile services at sex work hotspots [23–25]. However, the ideal model for HIV service provision to sex workers remains undetermined, and the strengths and weaknesses of current approaches are not fully understood. A recent cluster randomised control trial in Zimbabwe tested time to uptake of expanded of HIV services (including enhanced pre-exposure prophylaxis (PrEP) or ART initiation and adherence support) at sex work clinics through the inclusion of peer education outreach (standard of care versus more intense peer outreach services). The study found that while HIV testing and diagnosis improved between intervention and control arms, there was no significant difference in viral suppression [22].

With these challenges in mind, evidence from a large survey (2018) in three South African major cities found that while 73–87% of HIV-positive FSWs knew their status, only, 41–74% were currently taking ART [4]. Similarly, studies in Port Elizabeth and Soweto found 82% of HIV-positive FSWs knew their status, with ART uptake amongst 39% and 44% of them, respectively [18, 26]. The latter two South African studies pre-dated the WHO Universal Test and Treat (UTT) policy, and initiation of ART was only recommended for HIV-positive persons with a CD4 count < 200 cell/mm^3^ [27]. By September 2016, UTT was adopted within South Africa, offering all persons diagnosed HIV-positive to be initiated on treatment irrespective of CD4 + count [28]. These studies also pre-dated implementation of the South African National Sex Worker HIV Plan.

This study, therefore, aimed to describe the post-UTT state of the HIV CoC amongst FSWsin South Africa, and to explore potential impacts of sex work programmes on this CoC, after the implementation of the National Sex Worker HIV Plan 2016–2019, and prior to the SARS-Cov-2 pandemic, which has resulted in treatment interruptions across South Africa [29].

## Methods

### Study Design and Setting

The study was a cross-sectional national survey of FSWs conducted in South Africa in 2019 [30]. Study sites were located in 12 districts, across all nine South African provinces. The study was centrally coordinated with individual sites managed in partnership with non-government organisations (NGOs) running district-level sex work programmes. These organisations were all implementing programmes broadly in line with the National Sex Worker HIV Plan, although they did not provide the exact same set of services, and one site (in the Northern Cape) was a pilot programme with only peer-based services available. All sites had been operating in the sex work sector and providing services for more than 6 months at time of recruitment. Sites in major metropolitan areas such as Johannesbubrg, Cape Town, Ehkuruleni, Tshwane and Ethekwini offered a comprehensive set of HIV related services, whereas sites such as Vhembe, Francis Baard, and Ugu had new or pending comprehensive HIV services.

The services offered by all sites allowed for tracking of the HIV cascade of care targets. The minimum package of services available across all 12 sites were HIV testing and counseling, with antiretroviral treatment and management being available at 11 of the 12 sites. Additional services offered by most sites included: screening and syndromic management of sexually transmitted diseases, screening for tuberculosis, family planning, anthropometry assessments, vital sign screening, psychosocial support, viral load testing, PrEP initiation and routine bloods.

### Sampling

The overall study sample size was a minimum of 3000 FSWs, which was achieved using a multi-step sampling process [30]. Briefly, a stratified random sample of 12 districts was drawn from the 22/54 districts in South Africa that had active sex work programmes (ensuring ≥ one district per province). District sample sizes were proportional to FSW population size estimates [24]. All known sex work hotspots in the selected districts were mapped and categorised according to type (e.g. brothel, tavern, hotel, street, truck-stop). A stratified random sample of hotspots was drawn from each district to ensure representation of multiple hotspot types. Seed coupons (a voucher enabling screening and enrolment) were distributed at each randomly selected hotspot, and thereafter a chain referral method was used to enrol participants. Every participant successfully enrolled into the study was given three coupons and asked to distribute them at random to fellow FSWs who met the inclusion criteria. These were being a cis-gender female, ≥ 18 years, and having sold/transacted in sex in the preceding six months. For ethical reasons, being a current victim of human trafficking was an exclusion criterion [30]. This embedded study had a further inclusion criterion of HIV-positive.

### Data Collection

Data collection occured from February to June 2019. Data was collected directly into the Research Electronic Data Capture (REDCap) system [31] with built-in skip patterns and algorithms, on electronic tablets. The informed consent process and a comprehensive interviewer-administered questionnaire were conducted in a language of the participant’s choice. Peer interviewers (sex workers who underwent upskilling to ensure reliable data collection) were responsible for screening, consenting and main survey administration, which collected data on sociodemographics and sex work programme service uptake. Study nurses were responsible for a brief additional clinical survey, HIV testing, and phlebotomy in the case of an HIV indeterminate or positive result. Questions related to HIV testing history were asked in both interviewer- and nurse-administered surveys as a data quality-control check around participant self-reported HIV status disclosure. All blood samples were sent to the National Health Laboratory Service (NHLS) and to the National Institute of Communicable Diseases (NICD) for further testing, including viral load (VL).

### Study Measures

#### Sociodemographics

The sociodemographic data collected were place of birth and district of recruitment, age, migration status, highest level of education, and sex work history. Age in years was measured continuously, then categorised (18–24; 25–34; 35–44; 45+), as was sex work history (i.e.; age at entry into sex work: 10–17, 18–24, or 25+ ; and years in sex work: 1–2, 3–6, 7–14, or 15+) based on the interquartile ranges (IQR). Migrancy was defined as *local* if participants were working within their birth province, *internal immigrant* if South African but not working in their birth province, or *cross-border immigrant* if born outside of South Africa. Participants were questioned about their highest level of education achieved, and then responses were categorised as incomplete high school or complete high school.

### HIV Cascade of Care

HIV status was determined by the results of two, concurrent rapid HIV tests performed in the field. At the time of recruitment, study sites had three HIV rapid tests available for use: Abon™, First Response® and Toyo®. If rapid results were indeterminate a laboratory HIV Elisa was performed. Participant self-reported HIV testing history were one of the following: *no previous HIV test*, a *previous negative test*, a *previous positive test*, or a *previous indeterminate test*. A participant’s HIV positive status was defined as a *known positive,* if the participant self-reported a previous positive HIV test and tested HIV positive during the study; or as *newly diagnosed* if the participant did not previously know they were HIV-positive and tested HIV positive during the study.

Self-reported HIV treatment history responses included *no previous ART*, *previous ART but not currently on treatment*, or *currently on ART*. A participant’s ART status was defined as *on ART* if she self-reported initiating ART and there was no subsequent defaulting. Viral suppression was defined by dichotomising viral load results at 1000 copies/ml per South African clinical guidelines [32].

### Sex Worker Programme Services

A dichotomous variable was created to reflect whether an enrolment district had a well-established sex work programme that had been implementing comprehensive HIV services for at least six months prior to enrolment, or not. Participants selected from 30 specific services they received from sex work programmes in the previous six months. For this analysis, these services were collapsed into seven categories, as follows: *peer outreach* (seeing a sex work peer on outreach, receiving condoms or lubricant, receiving a health talk on outreach); *HIV testing or treatment* (being tested for HIV, being counselled on HIV status or adherence, accessing PrEP or ART, accessing prevention of mother-to-child transmission of HIV services (PMTCT)); *sexual and reproductive health* (sexually transmitted infection (STI) screening or treatment, contraception, cervical cancer screening or pap smears, breast exams); *“other” clinical services* (Tuberculosis (TB) screening or treatment, treatment of minor ailments, baby wellness checks or vaccinations); *mental health* (help with mental health problem, help with substance abuse problem, counselling for trauma or stress not related to HIV, attending general support groups); *violence or human rights violation* (help with a case of violence or human rights violation, post-rape care, legal assistance); and *financial and social* (seen a social worker, help with financial planning, help with ID/passport/birth certificate application, help with social grant application, help with nutritional support, help with opening a bank account, help with finding a place of safety).

### Ethical Considerations

Ethical approval was granted by the University of Witwatersrand Human Research Ethics Committee (HREC) (reference number 180809). Given the vulnerability of FSW populations, including the criminalisation of sex work in South Africa, ensuring the safety of participants was paramount. Embedding the study within existing partner organisations was included in the methodology partly to ensure ease of referral to medical treatment and psychosocial services.

The study team implemented strict safety protocols to ensure the protection and confidentiality of participants, these have been described in details in a previous methods publication (27). Specifically, to comply with ethical and human rights reasons, any person who was < 18 years old, or who self-reported being a victims of human trafficking, at time of recruitment, was excluded from enrolment in the study. When a study team member was notified of a child or victim of human trafficking, the site’s sex work programme was informed and their organisational specific protocols were implemented, including legal and psychosocial support (27).

### Statistical Analysis

Only the 1862 participants who tested HIV positive were included in this analysis. Preliminary analysis was conducted to assess missing data with < 5% missing in the overall database; none of the variables were missing > 10% of the data. Categorical variables were assessed by frequencies and proportions stratified by self-reported HIV status (known positive and newly diagnosed), self-reported ART (on ART vs. not on ART) and viral suppression on ART (suppressed vs. not suppressed), and bivariate associations were tested by Chi-square or Fisher’s exact test as appropriate; Chi-square (χ^2^) test statistics or Fishers exact test statistics are presented together with their associated p-values. If the global p-value for bivariate association was significant at 0.1 and the level of responses was > 2, we tested the null hypothesis of no difference between the stratas using the Chi-square test of proportions. Furthermore, sex work programme services were presented by age groups (18–24, 25–34, 35–44 and 45+ years), and comparisons between them conducted using bivariate association.

Factors associated with successful viral suppression were determined using a multivariate hierarchical logistic regression model where FSWs were nested in the main seed of each tree and the seeds were nested within sites. All variables were included in the model and adjusted for correlation using the unstructured covariance structure. The model was analysed using SAS Enterprise Guide 7.15 [33] using the SAS procedure GLIMMIX.

## Results

### HIV Cascade of Care

**Fig. 1:**
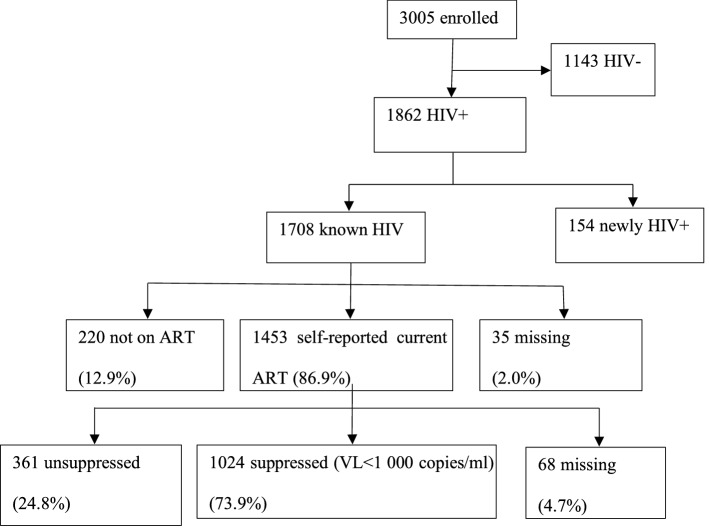
HIV Continuum of Care (CoC)

**Fig. 2:**
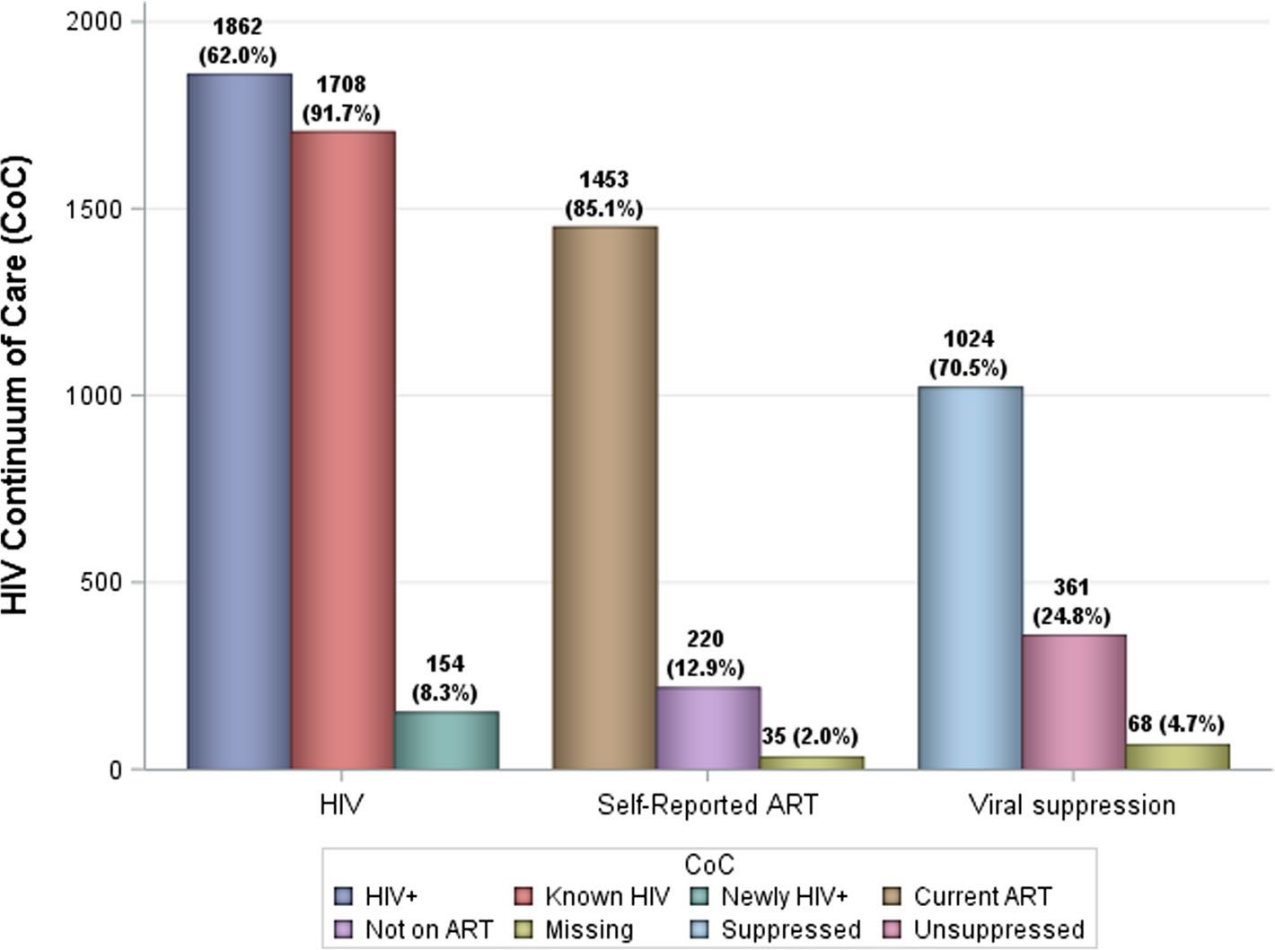
HIV Continuum of care for female sex workers towards the UN90-90–90 targets

Figures 1 and 2 depicts the HIV CoC. The overall HIV prevalence for the study population was 61.9% (27). This analysis included 1862 HIV-positive FSW participants. Of these, 91.7% (n = 1708/1862) self-reported being known positive, while 8.3% (n = 154/1862) were newly diagnosed. Of the known HIV-positive participants with ART history data available, 86.9% (n = 1453/1673) self-reported current ART use. Of those taking ART with VL data available, 73.9% (n = 1024/1385) were virally suppressed.

### Socio-Demographic Characteristics of HIV-Positive Female Sex Workers and the Cascade of Care

Of the total sample of HIV-positive FSW participants, almost half (45.7%, n = 850/1862) were between 25 and 34 years old, 62.3% (n = 1160/1862) were local sex workers, 16.3%, n = 303/1862) were cross-border immigrants. The majority of participants had not completed high school education (83.3%, n = 1548/1859), entered sex work above 24 years of age (50.4%, n = 930/1844) and had been involved in sex work for 3–6 years (38.3%, n = 711/1856) (Table 1).

**Table 1:** Sociodemographic characteristics of HIV positive FSWs and associations with HIV cascade of care indicators

Variables	Total	Self-report HIV status			Self-report ART status			Viral suppression on ART		
		Known positive	Newly diagnosed	P-Value (χ^2^)	On ART	Not on ART	P-Value (χ^2^)	Suppressed	Not suppressed	P-Value (χ^2^)
Age (in years)										
18–24	161/1862 (8.65)	141/1708 (8.26)^a^	20/154 (12.99)^a^	0.0872 (6.6)	108/1453 (7.43)^d^	29/220 (13.18)^d^	< .0001 (27.5)	61/1024 (5.96)^j^	44/361 (12.19)^j^	< .0001 (41.4)
25–34	850/1862 (45.65)	774/1708 (45.32)	76/154 (49.35)		632/1453 (43.50)^e^	122/220 (55.45)^e^		408/1024 (39.84)^k^	185/361 (51.25)^k^	
35–44	667/1862 (35.82)	621/1708 (36.36)	46/154 (29.87)		560/1453 (38.54)^f^	50/220 (22.73)^f^		429/1024 (41.89)^l^	112/361 (31.02)^l^	
45 +	184/1862 (9.88)	172/1708 (10.07)	12/154 (7.79)		153/1453 (10.53)	19/220 (8.64)		126/1024 (12.30)^m^	20/361 (5.54)^m^	
Migration										
Local	1160/1862 (62.30)	1063/1708 (62.24)	97/154 (62.99)	0.4691 (1.5)	891/1453 (61.32)^g^	152/220 (69.09)^g^	0.0707 (5.3)	598/1024 (58.40)^n^	246/361 (68.14)^n^	0.0028 (11.8)
Internal immigrant	399/1862 (21.43)	371/1708 (21.72)	28/154 (18.18)		323/1453 (22.23)	42/220 (19.09)		238/1024 (23.24)	71/361 (19.67)	
External immigrant	303/1862 (16.27)	274/1708 (16.04)	29/154 (18.83)		239/1453 (16.45)	26/220 (11.82)		188/1024 (18.36)^o^	44/361 (12.19)^o^	
Education										
Incomplete high school	1548/1859 (83.27)	1431/1705 (83.93)	117/154 (75.97)	0.0113 (6.4)	1209/1450 (83.38)	191/220 (86.82)	0.1967 (1.7)	841/1021 (82.37)	317/361 (87.81)	0.0159 (5.8)
Completed high school	311/1859 (16.73)	274/1705 (16.07)	37/154 (24.03)		241/1450 (16.62)	29/220 (13.18)		180/1021 (17.63)	44/361 (12.19)	
Age at entry into sex work										
10–17	198/1844 (10.74)	186/1690 (11.01)	12/154 (7.79)	0.4562 (1.6)	155/1437 (10.79)	26/219 (11.87)	< .0001 (22.1)	100/1014 (9.86)	43/355 (12.11)	0.0478 (6.1)
18–24	716/1844 (38.83)	653/1690 (38.64)	63/154 (40.91)		527/1437 (36.67)^h^	114/219 (52.05)^h^		355/1014 (35.01)	143/355 (40.28)	
25 +	930/1844 (50.43)	851/1690 (50.36)	79/154 (51.30)		755/1437 (52.54)^i^	79/219 (36.07)^i^		559/1014 (55.13)^p^	169/355 (47.61)^p^	
Years in sex work										
1–2	227/1856 (12.23)	195/1702 (11.46)^b^	32/154 (20.78)^b^	0.0006 (17.4)	157/1447 (10.85)	34/220 (15.45)	0.1336 (5.6)	104/1021 (10.19)	49/358 (13.69)	0.0007 (17.0)
3–6	711/1856 (38.31)	648/1702 (38.07)	63/154 (40.91)		544/1447 (37.60)	87/220 (39.55)		360/1021 (35.26)^q^	150/358 (41.90)^q^	
7–14	562/1856 (30.28)	519/1702 (30.49)	43/154 (27.92)		447/1447 (30.89)	62/220 (28.18)		320/1021 (31.34)	109/358 (30.45)	
15 +	356/1856 (19.18)	340/1702 (19.98)^c^	16/154 (10.39)^c^		299/1447 (20.66)	37/220 (16.82)		237/1021 (23.21)^r^	50/358 (13.97)^r^	

NB: P-value (χ^2^ test statistic) represents the global p-value comparison;

Where global p-values were significant, strata comparisons were conducted and were significant for the comparisons a-r

Self-report HIV status: a = 0.0454, b = 0.0007 and c = 0.0038; Self-report ART status: d = 0.0038, e = 0.0009, f < .0001, g = 0.0267, h < .0001 and i < .0001; Viral suppression on ART: j = 0.0001, k = 0.0002, l = 0.0003, m = 0.0003, n = 0.0011, o = 0.0069, p = 0.0145, q = 0.0251 and r = 0.0002

### Global Comparison

The proportion of known positives who did not complete high school was significantly higher (83.9% vs. 76.0%; p = 0.0113, χ^2^ = 6.4) than the newly diagnosed. Years in sex work (p = 0.0006, χ^2^ = 17.4) was associated with self-report HIV status (Table 1). Only age (p < 0.0001, χ^2^ = 27.5) and age entered into sex work (p < 0.0001, χ^2^ = 22.1) were associated with self-report ART. Viral suppression was associated with age (p < 0.0001, χ^2^ = 41.4), migration (p = 0.0028, χ^2^ = 11.8), age entered into sex work (p = 0.0478, χ^2^ = 6.1) and years in sex work (p = 0.0007, χ^2^ = 17.0). FSWs who were virally unsuppressed were more likely to have incomplete schooling (87.8% vs. 82.4%; p = 0.0159, χ^2^ = 5.8) compared with virally suppressed FSWs (Table 1).

A high proportion of newly diagnosed were recruited from a new or pending comprehensive HIV services site (65.6% vs. 46.9%; p < 0.0001, χ^2^ = 19.8), did not use HIV testing and treatment services (57.1% vs. 42.9%; p = 0.0007, χ^2^ = 11.6), did not use sexual and reproductive health services (66.9% vs. 54.2%; p = 0.0025, χ^2^ = 9.2), did not use any other clinical services (90.9% vs. 83.1%; p = 0.0122, χ^2^ = 6.3), did not use mental health services (82.5% vs. 73.3%; p = 0.0129, χ^2^ = 6.2) and did not use violence or human rights violation services (89.0% vs. 80.4%; p = 0.0092, χ^2^ = 6.8) compared to the known positive female sex workers. None or one sex work programme service (p = 0.0006, χ^2^ = 19.4) was associated with self-report HIV status (Table 2).

**Table 2:** Sex work programme service use among HIV positive FSWs and associations with HIV cascade of care indicators

		Self-report HIV status			Self-report ART status			Viral suppression on ART		
Variables	Total	Known positive	Newly diagnosed	P-value (χ^2^)	On ART	Not on ART	P-value (χ^2^)	Suppressed	Not suppressed	P-value (χ^2^)
Site of recruitment										
New (< 6 months) or pending comprehensive HIV services	902/1862 (48.44)	801/1708 (46.90)	101/154 (65.58)	< .0001 (19.8)	673/1453 (46.32)	112/220 (50.91)	0.2035 (1.6)	458/1024 (44.73)	181/361 (50.14)	0.0761 (3.1)
Established comprehensive HIV services (> 6 months)	960/1862 (51.56)	907/1708 (53.10)	53/154 (34.42)		780/1453 (53.68)	108/220 (49.09)		566/1024 (55.27)	180/361 (49.86)	
Use of peer outreach services										
No	108/1862 (5.80)	99/1708 (5.80)	9/154 (5.84)	0.9806 (0.0)	87/1453 (5.99)	11/220 (5.00)	0.5610 (0.3)	68/1024 (6.64)	16/361 (4.43)	0.1306 (2.3)
Yes	1754/1862 (94.20)	1609/1708 (94.20)	145/154 (94.16)		1366/1453 (94.01)	209/220 (95.00)		956/1024 (93.36)	345/361 (95.57)	
Use of HIV testing and treatment services										
No	821/1862 (44.09)	733/1708 (42.92)	88/154 (57.14)	0.0007 (11.6)	614/1453 (42.26)	100/220 (45.45)	0.3716 (0.8)	411/1024 (40.14)	165/361 (45.71)	0.0649 (3.4)
Yes	1041/1862 (55.91)	975/1708 (57.08)	66/154 (42.86)		839/1453 (57.74)	120/220 (54.55)		613/1024 (59.86)	196/361 (54.29)	
Use of sexual and reproductive health services										
No	1029/1862 (55.26)	926/1708 (54.22)	103/154 (66.88)	0.0025 (9.2)	789/1453 (54.30)	114/220 (51.82)	0.4910 (0.5)	557/1024 (54.39)	200/361 (55.40)	0.7410 (0.1)
Yes	833/1862 (44.74)	782/1708 (45.78)	51/154 (33.12)		664/1453 (45.70)	106/220 (48.18)		467/1024 (45.61)	161/361 (44.60)	
Use of any other clinical services										
No	1560/1862 (83.78)	1420/1708 (83.14)	140/154 (90.91)	0.0122 (6.3)	1212/1453 (83.41)	181/220 (82.27)	0.6727 (0.2)	872/1024 (85.16)	287/361 (79.50)	0.0124 (6.3)
Yes	302/1862 (16.22)	288/1708 (16.86)	14/154 (9.09)		241/1453 (16.59)	39/220 (17.73)		152/1024 (14.84)	74/361 (20.50)	
Use of mental health services										
No	1379/1862 (74.06)	1252/1708 (73.30)	127/154 (82.47)	0.0129 (6.2)	1061/1453 (73.02)	166/220 (75.45)	0.4469 (0.6)	747/1024 (72.95)	264/361 (73.13)	0.9469 (0.0)
Yes	483/1862 (25.94)	456/1708 (26.70)	27/154 (17.53)		392/1453 (26.98)	54/220 (24.55)		277/1024 (27.05)	97/361 (26.87)	
Use of assistance with violence or human rights violations										
No	1510/1862 (81.10)	1373/1708 (80.39)	137/154 (88.96)	0.0092 (6.8)	1170/1453 (80.52)	175/220 (79.55)	0.7336 (0.1)	830/1024 (81.05)	288/361 (79.78)	0.5971 (0.3)
Yes	352/1862 (18.90)	335/1708 (19.61)	17/154 (11.04)		283/1453 (19.48)	45/220 (20.45)		194/1024 (18.95)	73/361 (20.22)	
Use of other social and financial assistance										
No	1609/1862 (86.41)	1470/1708 (86.07)	139/154 (90.26)	0.1457 (2.1)	1250/1453 (86.03)	190/220 (86.36)	0.8937 (0.0)	878/1024 (85.74)	314/361 (86.98)	0.5591 (0.3)
Yes	253/1862 (13.59)	238/1708 (13.93)	15/154 (9.74)		203/1453 (13.97)	30/220 (13.64)		146/1024 (14.26)	47/361 (13.02)	
Number of SW programme services used										
0–1	531/1862 (28,52)	469/1708 (27.45)^a^	62/154 (40.26)^a^	0.0006 (19.4)	391/1453 (26,91)	66/220 (30.00)	0.4164 (1.7)	281/1024 (27.44)	89/361 (24.65)	0.5394 (1.2)
2–3	796/1862 (42.75)	727/1708 (42.56)	69/154 (44.81)		628/1453 (43.22)	85/220 (38.64)		440/1024 (42.97)	165/361 (45.71)	
4 +	535/1862 (28.73)	512/1708 (29.98)^b^	23/154 (14.94)^b^		434/1453 (28.87)	69/220 (31.36)		303/1024 (29.59)	107/361 (29.64)	

NB: P-value (χ^2^ test statistic) represents the global p-value comparison;

where global p-values were significant, strata comparisons were conducted and were significant for the comparisons a and b

Self-report HIV status: ^a^0.0007 and ^b^0.0038

There was no significant difference in service utilisation for those on ART compared to those not on ART. Compared to virally unsuppressed female sex workers, a higher proportion of virally suppressed did not report using other clinical services (85.2% vs. 79.5%; p = 0.0124, χ^2^ = 6.3) (Table 2).

### Strata Comparison

The proportion of known positives who sold sex for at least 15 years (20.0% vs. 10.4%; p = 0.0038) was higher than that of the newly diagnosed. A higher proportion of newly diagnosed HIV positives had been involved in sex work for 1–2 years (20.8% vs. 11.5%; p = 0.0007) compared to the known positive (Table 1).

Relative to FSWsnot on ART, those on ART were likely to be 35–44 years old (38.5% vs. 22.7%; p < 0.0001) and to have entered sex work over the age of 24 years (52.1% vs. 36.7%; p < 0.0001). However, there was a higher proportion not on ART in 18–24 (13.2% vs. 7.4%; p = 0.0038) and 25–34 year olds (55.5% vs. 43.5%; p = 0.0009), local FSWs(69.1% vs. 61.3%; p = 0.0267), and had entered sex work at 18–24 years (52.5% vs. 36.1%; p < 0.0001) relative to those on ART (Table 1).

A higher proportion of virally suppressed FSWswere 35–44 (41.9% vs. 31.0%; p = 0.0003) and 45 + year olds (12.3% vs. 5.5%; p = 0.0003), external immigrants (18.4% vs. 12.2%; p = 0.0069), entered sex work above 24 years of age (55.1% vs. 47.6%; p = 0.0145) and had been in sex work for at least 15 years (23.2% vs. 14.0%; p = 0.0002) compared to those who were virally unsuppressed (Table 1). On the other hand, those who were virally unsuppressed were more likely to be 18–24 (12.2% vs. 6.0%; p = 0.0001) and 25–34 years old (51.3% vs. 39.8%; p = 0.0002), local FSWs(68.1% vs. 58.4%; p = 0.0011) and involved in sex work for 3–6 years (41.9% vs. 35.3%; p = 0.0251) compared with virally suppressed FSWs (Table 1).

A high proportion of newly diagnosed used none or one sex work programme service (40.3% vs. 27.5%; p = 0.0008) compared to the known positive FSWs(Table 2).

There were no significant differences in service uptake by age group (Supplementary Table 1).

### Factors Associated with Viral Suppression

In the hierarchical multivariate regression (Table 3), there was a higher likelihood of being virally supressed in 25–34 (OR 1.6, 95% CI 1.04–2.61), 35–44 (OR 2.8, 95% CI 1.7–4.5) and 45 + year olds (OR 5.2, 95% CI 2.7–9.9), external immigrants (OR 1.5, 95% CI 1.03–2.2) and among those who utilised HIV testing and treatment services (OR 1.4, 95% CI 1.1–1.8). The likelihood of viral supression was lower among those utilising other clinical services (OR 0.6, 95% CI 0.4–0.8).

**Table 3:** Factors associated with viral suppression in HIV positive FSWs on ART

Variable	Adjusted odds ratio	95% confidence interval	P-value
Age category			
18–24	Ref	–	–
25–34	1.6	(1.04–2.61)	0.032
35–44	2.8	(1.7–4.5)	< 0.001
45 +	5.2	(2.7–9.9)	< 0.001
Migration			
Local	Ref	–	–
Internal immigrant	1.2	(0.8–1.6)	0.361
External immigrant	1.5	(1.03–2.2)	0.033
Education			
Incomplete high school	Ref	–	–
Completed high school	1.4	(1.0–2.0)	0.084
Age at entry into sex work			
10–17	Ref	–	–
18–24	1.0	(0.6–1.5)	0.953
25 +	0.9	(0.6–1.4)	0.680
Site of recruitment			
New (< 6 months) or pending comprehensive HIV services	Ref	–	–
Established comprehensive HIV services (> 6 months)	1.1	(0.9–1.5)	0.377
Sex work programme service utilisation			
Received HIV testing or treatment in the past 6 months			
No	Ref	–	–
Yes	1.4	(1.1–1.8)	0.016
Received other clinical services in the past 6 months			
No	Ref	–	–
Yes	0.6	(0.4–0.8)	0.003

## Discussion

This study presents the first national evidence on the HIV cascade amongst FSWs in South Africa, sampling from all nine provinces. The HIV CoC showed 92% of the total FSWs in our sample knew their HIV positive status, 85% of these self-reported that they were currently on ART, and 71% of those on ART were virally suppressed. These findings regarding the HIV CoC are an improvement compared to both previous HIV CoC amongst the FSW population and the general population, particularly with respect to the first two indicators (known positivity and ART coverage) [3, 34]. FSWs who engaged in multiple services within specialised sex work programmes were more likely to know their HIV status, suggesting a broader range of integrated, healthcare systems serve as stronger entry points into the HIV CoC compared to the current vertical structure of the HIV systems (HIV testing, ART initiation and viral load monitoring). The finding that only three quarters of FSWs on ART were virally supressed is significantly lower than amongst the general population and cause for concern.

The evidence from this study affirms the work of specialised sex work programmes and suggests the HIV CoC is significantly improved in this setting, particularly with respect to known HIV status and ART use. As compared to the HIV CoC studies pre-dating UTT and sex work programmes conducted in major cities, this study reports an increase of 5–35% of HIV-positive FSWs knowing their HIV status, and of those a 42–64% increase in ART use across different geolocations [4, 18, 26]. Overall our study had somewhat stronger rates for known HIV status and ART use compared to the 2018 South African Health Monitoring Survey(SAHMS) [4] among female sex workers. A comparison between the two studies by the three major metropolitans, suggests that ART status is comparable between Cape Town, with our findings being within the lower CI ranges for Johannesburg and Ethekwini of the SAHMS. Compared to data from the South African general population, post-dating UTT, reporting 84% of 15–64 year olds living with HIV know their status, of which 71% have initiated ART, and 86% exhibit viral suppression) [34]; our study also reports a stronger HIV CoC for the first two indicators. However, our study had similar rates of known positives initiating ART as a recent linkage to care study conducted at an integrated non-communicable disease—HIV testing centre at PHRU for adults (18 + years) from the general population of Soweto (88.6%) [35].

Engagement in multiple, integrated services (including HIV testing, sexual and reproductive health, mental health, and assistance with post-violence care) was linked to a greater likehihood of HIV positive FSWsknowing their status. The more services utilised, the higher the likelihood that an HIV-positive FSW would know her status. This suggests the need to ensure that services offered are matched to the needs of the population, and strategically used to draw sex workers into sex work programme activities. The multivariable logistic regression analysis also demonstrated that receiving HIV testing and treatment services through specialised sex work programmes significantly improved the odds of viral suppression, by 1.4 times, compared to HIV-positive FSWs who were not receiving these services through a sex work programme.

Our findings strongly support and reaffirm the need for a special focus on the risk and burden of HIV in adolescent girls and young women (AGYW) who sell sex [14, 15]. Population-based surveys from South Africa indicate that while 16% of women between the ages of 15–24 years are HIV-positive, only 19%, respectively, within this group were aware of their HIV-positive status [36]. Standard of care HIV testing is primarily facility-based, and there is continued low uptake of testing amongst adolescent girls and young women, despite their high level of awareness and willingness to test for HIV [37, 38]. Half of our study population began selling sex as adolescent girls and young women, and FSWscurrently falling into the age category of 18–24 years of age had substantially poorer CoC indicators than FSWsof older age and younger age was a significant contributor to being virally unsupressed. They were less likely to know their status or to report being on current ART, and crucially, less likely to be virally suppressed. These findings regarding younger FSWscorroborate previous research in the African region [19, 20]. Since fewer years in sex work was also correlated with poorer viral suppression. Programmes should consider young, new FSWsas particularly vulnerable to virological failure, and likely to require more intensive treatment support [38]. The reasons for low rates of viral suppression needs to be investigated, and effective interventions implemented to address barriers to adherence in female sex workers.

The overall improved HIV CoC amongst FSWsin this study could be attributed to the model of service, which includes a peer-led and community-based approach, flexible operational hours, integrated services, and a sensitised method of service provision. Each of these factors has an established evidence base towards improved uptake of HIV services [6, 16, 17, 20, 22–25]. Our study compliments the previous evidence, as it has also shown an improved HIV CoC. However, the success of these interventions seem to have made a lesser impact on younger age groups and/or amongst FSWswho have been working in the sector for less time, as well as on successful viral suppression. With FSWage not being significantly associated with uptake of services, it is currently unclear why this is. With the expiry of the National Sex Worker HIV Plan 2016–2019, and the late release of South Africa’s National Sex Worker HIV, TB and STI Plan (2019–2022) [7], it is critical to further investigate gaps, so that future iteration of sex work programmes may produce even stronger health-related outcomes. This will become even more important should the impact of the COVID-19 pandemic have resulted in poorer health outcomes overall amongst sex workers.

It is a strength of this research that we have managed to implement a rigorous study with a fairly representative sample of FSWsacross South Africa working in areas linked to programmes. We worked with FSWsas partners and collaborated on questionnaire development, implementation and data collection. This was important given the stigma and vulnerability that FSWsface.

We are not aware of a previous successful attempt to implement a cross sectional, national level study with FSWsin South Africa. Care should be taken when interpreting findings, as sex workers recruited were within provinces with existing sex work friendly services. Other strengths of the study included the efficient nature of the design to collect the required data and its ability to access a large sample size over a short period of time with a wide coverage and diversity across locations. This was achieved by collaborating with established sites where sex workers have had access to services for some time. However, we did not include any sites that were not receiving donor funding and disricts with no sex work programmes are unlikely to have the successes in achieving the CoC as seen in this study. Previous HIV test results and ART uptake are self-reported, and therefore could be under- or over-reported by participants due to social desirability bias. However, it is our opinion that these findings represent real and meaningful shifts in CoC in the FSWpopulation over the past years.

## Conclusions and Recommendations

These study findings present strikingly impressive gains with respect to achieving the WHO 90:90:90, and largely aligned to the 2018 SAHMS, reviewing targets amongst FSWsin South Africa in recent years. This is likely aided in considerable part by sex worker-friendly programmes, operating in line with the government’s National Sex Worker HIV Plans since 2016 [4, 39], and critically important given the criminalisation of sex work and prevalence of discrimination against sex workers. This study highlights the slower progress towards viral suppression amongst female sex workers, as compared to the first two CoC indicators. Further research is needed to understand barriers towards suppression and the tailored interventions required for this population. Specifically, and while the South African National Sex Worker Plan (2019–2022) makes brief mention of monitoring PrEP uptake amongst AGYW [7], the unique needs of AGYW sex workers must be addressed to ensure the risk of slipping between the gaps of sex work and AGYW programmes are minimised. Our evidence pre-dates the COVID-19 pandemic, and there is concern that changes to health-seeking behaviour and interruptions to diagnostic and treatment services may have negatively affected CoC indicators. Research to assess the impact of the pandemic on the HIV CoC in sex workers and other key populations would be greatly welcomed.

## Supplementary Information

Below is the link to the electronic supplementary material.

Supplementary file1 (DOCX 24 kb)

Supplementary file2 (XLSX 697 kb)

## Data Availability

Data will be made publically available through the journals author share portal. For the purpose of open access, the author has applied a CC BY public copyright licence to any Author Accepted Manuscript version arising from this submission.
