# Supplementary material for: The HIV Cascade of Care and Service Utilisation at Sex Work Programmes Among Female Sex Workers in South Africa
Source: AIDS Behav. 2022 Mar 5;26(9):2907–19. doi: 10.1007/s10461-022-03616-6 (PMC8897612; doi:10.1007/s10461-022-03616-6)
Supplement: Supplementary file 1 — Supplementary file1 (DOCX 24 kb) [file 10461_2022_3616_MOESM1_ESM.docx]

**Supplementary Table 1: Sex work programme service use among HIV positive FSWs by age**

| **Variables** | **Total (n=1385)** | **18-24 (n=105)** | **25-34 (n=593)** | **35-44 (n=541)** | **45+**  **(n=146)** | **P-Value (χ^2^)** |
| --- | --- | --- | --- | --- | --- | --- |
|  |  |  |  |  |  |  |
| **Use of peer outreach services** |  |  |  |  |  |  |
| No | 84/1385 (6.06) | 5/105 (4.76) | 29/593 (4.89) | 36/541 (6.65) | 14/146 (9.59) | 0.1536 (5.3) |
| Yes | 1301/1385 (93.94) | 100/105 (95.24) | 564/593 (95.11) | 505/541 (93.35) | 132/146 (90.41) |  |
|  |  |  |  |  |  |  |
| **Use of HIV testing and treatment services** |  |  |  |  |  |  |
| No | 576/1385 (41.59) | 43/105 (40.95) | 245/593 (41.32) | 216/541 (39.93) | 72/146 (49.32) | 0.2368 (4.2) |
| Yes | 809/1385 (58.41) | 62/105 (59.05) | 348/593 (58.68) | 325/541 (60.07) | 74/146 (50.68) |  |
|  |  |  |  |  |  |  |
| **Use of sexual and reproductive health services** |  |  |  |  |  |  |
| No | 757/1385 (54.66) | 59/105 (56.19) | 320/593 (53.96) | 291/541 (53.79) | 87/146 (59.59) | 0.6123 (1.8) |
| Yes | 628/1385 (45.34) | 46/105 (43.81) | 273/593 (46.04) | 250/541 (46.21) | 59/146 (40.41) |  |
|  |  |  |  |  |  |  |
| **Use of any other clinical services** |  |  |  |  |  |  |
| No | 1159/1385 (83.68) | 91/105 (86.67) | 491/593 (82.80) | 456/541 (84.29) | 121/146 (82.88) | 0.7438 (1.2) |
| Yes | 226/1385 (16.32) | 14/105 (13.33) | 102/593 (17.20) | 85/541 (15.71) | 25/146 (17.12) |  |
|  |  |  |  |  |  |  |
| **Use of mental health services** |  |  |  |  |  |  |
| No | 1011/1385 (73.00) | 83/105 (79.05) | 442/593 (74.54) | 380/541 (70.24) | 106/146 (72.60) | 0.1902 (4.8) |
| Yes | 374/1385 (27.00) | 22/105 (20.95) | 151/593 (25.46) | 161/541 (29.76) | 40/146 (27.40) |  |
|  |  |  |  |  |  |  |
| **Use of assistance with violence or human rights violations** |  |  |  |  |  |  |
| No | 1118/1385 (80.72) | 90/105 (85.71) | 476/593 (80.27) | 434/541 (80.22) | 118/146 (80.82) | 0.6047 (1.8) |
| Yes | 267/1385 (19.28) | 15/105 (14.29) | 117/593 (19.73) | 107/541 (19.78) | 28/146 (19.18) |  |
|  |  |  |  |  |  |  |
| **Use of other social and financial assistance** |  |  |  |  |  |  |
| No | 1192/1385 (86.06) | 96/105 (91.43) | 510/593 (86.00) | 459/541 (84.84) | 127/146 (86.99) | 0.3480 (3.3) |
| Yes | 193/1385 (13.94) | 9/105 (8.57) | 83/593 (14.00) | 82/541 (15.16) | 19/146 (13.01) |  |
|  |  |  |  |  |  |  |

**NB:  P-value (**χ^2^ **test statistic) represents the global p-value comparison;**

**Supplementary Table 2: Demographics and outcomes by site**

| **Variables** | **Bojanala** | **Buffalo City** | **Cape Town** | **Johannesburg** | **Ekurhuleni** | **EThekwini** | **Francis Baard** | **Thabo Mofutsanyane** | **Tshwane** | **Ugu** | **Vhembe** |
| --- | --- | --- | --- | --- | --- | --- | --- | --- | --- | --- | --- |
|  |  |  |  |  |  |  |  |  |  |  |  |
| **Age (in years)** |  |  |  |  |  |  |  |  |  |  |  |
| 18-24 | 9/190 (4.74) | 15/170 (8.82) | 3/96 (3.13) | 33/376 (8.78) | 3/86 (3.49) | 32/301 (10.63) | 7/55 (12.73) | 18/139 (12.95) | 3/101 (2.97) | 11/65 (16.92) | 6/95 (6.32) |
| 25-34 | 76/190 (40.00) | 67/170 (39.41) | 54/96 (56.25) | 161/376 (42.82) | 34/86 (39.53) | 144/301 (47.84) | 29/55 (52.73) | 74/139 (53.24) | 34/101 (33.66) | 36/65 (55.38) | 37/95 (38.95) |
| 35-44 | 84/190 (44.21) | 60/170 (35.29) | 36/96 (37.50) | 146/376 (38.83) | 45/86 (52.33) | 96/301 (31.89) | 17/55 (30.91) | 38/139 (27.34) | 45/101 (44.55) | 14/65 (21.54) | 37/95 (38.95) |
| 45+ | 21/190 (11.05) | 28/170 (16.47) | 3/96 (3.13) | 36/376 (9.57) | 4/86 (4.65) | 29/301 (9.63) | 2/55 (3.64) | 9/139 (6.47) | 19/101 (18.81) | 4/65 (6.15) | 15/95 (15.79) |
|  |  |  |  |  |  |  |  |  |  |  |  |
| **Migration** |  |  |  |  |  |  |  |  |  |  |  |
| Local | 50/190 (26.32) | 167/170 (98.24) | 58/96 (60.42) | 169/376 (44.95) | 2/86 (2.33) | 267/301 (88.70) | 23/55 (41.82) | 128/139 (92.09) | 37/101 (36.63) | 60/65 (92.31) | 65/95 (68.42) |
| Internal immigrant | 57/190 (30.00) | 2/170 (1.18) | 33/96 (34.38) | 158/376 (42.02) | 9/86 (10.47) | 32/301 (10.63) | 17/55 (30.91) | 7/139 (5.04) | 45/101 (44.55) | 5/65 (7.69) | 1/95 (1.05) |
| External immigrant | 83/190 (43.68) | 1/170 (0.59) | 5/96 (5.21) | 49/376 (13.03) | 75/86 (87.21) | 2/301 (0.66) | 15/55 (27.27) | 4/139 (2.88) | 19/101 (18.81) | 0/65 (0.00) | 29/95 (30.53) |
|  |  |  |  |  |  |  |  |  |  |  |  |
| **Education** |  |  |  |  |  |  |  |  |  |  |  |
| Incomplete high school | 144/190 (75.79) | 144/170 (84.71) | 83/96 (86.46) | 324/376 (86.17) | 42/86 (48.84) | 260/299 (86.96) | 43/55 (78.18) | 117/139 (84.17) | 83/101 (82.18) | 56/64 (87.50) | 79/95 (83.16) |
| Complete high school | 46/190 (24.21) | 26/170 (15.29) | 13/96 (13.54) | 52/376 (13.83) | 44/86 (51.16) | 39/299 (13.04) | 12/55 (21.82) | 22/139 (15.83) | 18/101 (17.82) | 8/64 (12.50) | 16/95 (16.84) |
|  |  |  |  |  |  |  |  |  |  |  |  |
| **Self-report HIV status** |  |  |  |  |  |  |  |  |  |  |  |
| Known positive | 168/190 (88.42) | 144/170 (84.71) | 91/96 (94.79) | 347/376 (92.29) | 77/86 (89.53) | 292/301 (97.01) | 53/55 (96.36) | 132/139 (94.96) | 100/101 (99.01) | 63/65 (96.92) | 68/95 (71.58) |
| Newly diagnosed | 22/190 (11.58) | 26/170 (15.29) | 5/96 (5.21) | 29/376 (7.71) | 9/86 (10.47) | 9/301 (2.99) | 2/55 (3.64) | 7/139 (5.04) | 1/101 (0.99) | 2/65 (3.08) | 27/95 (28.42) |
|  |  |  |  |  |  |  |  |  |  |  |  |
| **Self-report ART status** |  |  |  |  |  |  |  |  |  |  |  |
| No | 18/166 (10.84) | 16/136 (11.76) | 21/80 (26.25) | 48/342 (14.04) | 3/74 (4.05) | 30/292 (10.27) | 13/50 (26.00) | 19/132 (14.39) | 6/100 (6.00) | 4/63 (6.35) | 15/66 (22.73) |
| Yes | 148/166 (89.16) | 120/136 (88.24) | 59/80 (73.75) | 294/342 (85.96) | 71/74 (95.95) | 262/292 (89.73) | 37/50 (74.00) | 113/132 (85.61) | 94/100 (94.00) | 59/63 (93.65) | 51/66 (77.27) |
|  |  |  |  |  |  |  |  |  |  |  |  |
| **Viral suppression on ART** |  |  |  |  |  |  |  |  |  |  |  |
| Suppressed | 113/145 (77.93) | 72/115 (62.61) | 31/41 (75.61) | 218/288 (75.69) | 58/70 (82.86) | 184/258 (71.32) | 25/35 (71.43) | 80/107 (74.77) | 75/89 (84.27) | 41/55 (74.55) | 33/47 (70.21) |
| Unsuppressed | 32/145 (22.07) | 43/115 (37.39) | 10/41 (24.39) | 70/288 (24.31) | 12/70 (17.14) | 74/258 (28.68) | 10/35 (28.57) | 27/107 (25.23) | 14/89 (15.73) | 14/55 (25.45) | 14/47 (29.79) |
|  |  |  |  |  |  |  |  |  |  |  |  |

**Supplementary Table 3: Factors associated with viral unsuppressed in HIV positive FSWs on ART**

| **Variables** | **Adjusted Odds Ratio** | **95% Confidence Interval** | **P-Value** |
| --- | --- | --- | --- |
|  |  |  |  |
| **Age category** |  |  |  |
| 18-24 | ref | - | - |
| 25-34 | 0.6 | (0.4-0.95) | 0.032 |
| 35-44 | 0.3 | (0.2-0.6) | <.0001 |
| 45+ | 0.2 | (0.1-0.4) | <.0001 |
| **Migration** |  |  |  |
| **Local** | ref | - | - |
| Internal immigrant | 0.9 | (0.6-1.2) | 0.362 |
| External immigrant | 0.7 | (0.4-0.96) | 0.033 |
| **Education** |  |  |  |
| Incomplete high school | ref | - | - |
| Completed high school | 0.7 | (0.5-1.0) | 0.084 |
| **Age at entry into sex work** |  |  |  |
| 10-17 | ref | - | - |
| 18-24 | 1.0 | (0.7-1.5) | 0.953 |
| 25+ | 1.1 | (0.7-1.7) | 0.680 |
| **Site of recruitment** |  |  |  |
| New (<6 months) or pending comprehensive HIV services | ref | - | - |
| Established comprehensive HIV services (>6 months) | 0.9 | (0.7-1.2) | 0.379 |
| **Sex work programme service utilisation** |  |  |  |
| **Received HIV testing or treatment in the past 6 months** |  |  |  |
| No HIV testing or treatment | ref | - | - |
| HIV testing and treatment | 0.7 | (0.5-0.9) | 0.017 |
|  |  |  |  |
| **Received other clinical services in the past 6 months** |  |  |  |
| No other clinical services | ref | - | - |
| Other clinical services | 1.7 | (1.2-2.3) | 0.003 |
|  |  |  |  |
